# Functional Status and Postoperative Outcomes After Left Pancreatectomy for Pancreatic Neuroendocrine Tumors: An Exploratory Analysis from the Prospective SPANDISPAN Study †

**DOI:** 10.3390/jcm15187035

**Published:** 2026-09-11

**Authors:** José Manuel Ramia, Belén Hernández-Roca, Juan Jesús Rubio-García, Daniel Aparicio-López, Juli Busquets, Luis Secanella, Nuria Peláez, Maialen Alkorta, Itziar de Ariño-Hervás, María del Mar Achalandabaso, Enrique Toledo, Fernando Rotellar, Pablo Martí-Cruchaga, Miguel Ángel Gómez-Bravo, Gonzalo Suárez, Marina Garcés-Albir, Luis Sabater, Gabriel García, Francisco Javier Alcalá, Enrique Asensio, David Pacheco, Esteban Cugat, Francisco Espín, María Galófre, Belinda Sánchez, Julio Santoyo, Jorge Calvo, Carmelo Loinaz, María Isabel García-Domingo, Santiago Sánchez-Cabús, Belén Martín, Gerardo Blanco-Fernández, Isabel Jaén-Torrejimeno, Carlos Domingo, Carmen Payá, Carmen González, Sandra Ruiz, Rafael López-Andújar, Cristina Ballester, Ana Belén Vico, Natalia Zambudio, Sergio Estévez, Manuel Nogueira-Sixto, José Ignacio Miota, Belén Conde, Miguel Ángel Suárez, Jorge Roldán de la Rúa, Angélica Blanco, Manuel González, Pilar Elena González, Betsabé Reyes, Santiago López-Ben, Berta Tió, Javier Mínguez, Inmaculada Lasa, Alberto Miyar, Lorena Solar, Fernando Burdío, Benedetto Ielpo, Alberto Carabias, María Paloma Sanz-Muñoz, Alfredo Escartín, Fulthon Vela, Elia Marqués, Adelino Pérez, Gloria Palomares, Antonio Calvo, José Tomás Castell, María Jesús Castro, María del Carmen Manzanares, Enrique Artigues, Juan Luis Blas, Luis Díez, Alicia Calero, José E. Quiñones, Mario Rodríguez, Mario Serradilla-Martín, Cándido F. Alcázar-López, Celia Villodre

**Affiliations:** 1 Department of Surgery, General University Hospital Dr. Balmis, 03010 Alicante, Spaincandidoalcazar@hotmail.com (C.F.A.-L.); celivii@gmail.com (C.V.); 2 Institute of Health and Biomedical Research of Alicante, Instituto de Investigación Sanitaria y Biomédica de Alicante (ISABIAL), 03010 Alicante, Spain; 3 Department of Surgery, School of Medicine, Miguel Hernández University, 03202 Alicante, Spain; 4 Department of Surgery, Hospital General Universitario de Elda, 03600 Alicante, Spain; belenhdezr95@gmail.com; 5 Department of Surgery, Hospital Ernest Lluch, 50300 Calatayud, Spain; 6 Department of Surgery, Hospital Universitario Bellvitge, 08907 L’Hospitalet de Llobregat, Spain; 7 Department of Surgery, Hospital Universitario de Donostia, 20014 San Sebastián, Spain; 8 Department of Surgery, Hospital Universitario Marqués de Valdecilla, 39008 Santander, Spain; 9 Department of Surgery, Clínica Universidad de Navarra, 31008 Pamplona, Spain; 10 Department of Surgery, Hospital Universitario Virgen del Rocío, 41013 Sevilla, Spain; 11 Liver-Biliary and Pancreatic Unit, Department of General and Digestive Surgery, Hospital Clinico Universitario, Biomedical Research Institute, INCLIVA, 46010 Valencia, Spain; marinagarcesalbir@gmail.com (M.G.-A.);; 12 Department of Surgery, Hospital Insular de Gran Canaria, 35016 Las Palmas de Gran Canaria, Spain; 13 Department of Surgery, Hospital Universitario Rio Ortega, 47012 Valladolid, Spain; 14 Department of Surgery, Hospital Universitario German Trias i Pujol, 08916 Badalona, Spain; 15 Department of Surgery, Hospital Regional Universitario Carlos Haya, 29010 Málaga, Spain; 16 Department of Surgery, Hospital Universitario Doce de Octubre, 28041 Madrid, Spain; 17 Department of Surgery, Hospital Universitario Mutua de Terrassa, 08221 Terrassa, Spain; 18 Department of Surgery, Hospital Universitario Santa Creu i Sant Pau, 08025 Barcelona, Spain; 19 Department of Surgery, Hospital Universitario de Badajoz, 06080 Badajoz, Spainisabel.jaen@salud-juntaex.es (I.J.-T.); 20 Department of Surgery, Hospital Universitario Doctor Peset, 56017 Valencia, Spain; 21 Department of Surgery, Hospital Universitario de Basurto, 48013 Bilbao, Spain; 22 Surgery HPB and Liver Transplant Unit, Instituto de Investigación Sanitaria La Fe (IIS La Fe) & CIBEREHD, Universitary and Politecnic Hospital La Fe, 46026 Valencia, Spain; 23 Department of Surgery, Hospital Universitario Virgen de las Nieves, 18012 Granada, Spain; 24 Department of Surgery, Complejo Hospitalario Universitario de Vigo, 36312 Vigo, Spain; 25 Department of Surgery, Hospital General Universitario de Albacete, 02008 Albacete, Spain; 26 Department of Surgery, Hospital Universitario Virgen de la Victoria, 29010 Málaga, Spain; 27 Department of Surgery, Complejo Hospitalario Universitario Juan Canalejo, 15006 A Coruña, Spain; 28 Department of Surgery, Hospital Universitario Nuestra Sra. de la Candelaria, 38010 Tenerife, Spain; 29 Department of Surgery, Hospital Universitario Josep Trueta, 17007 Girona, Spain; slopezben.girona.ics@gencat.cat (S.L.-B.);; 30 Department of Surgery, Hospital Universitario Príncipe de Asturias, 28805 Alcalá de Henares, Spain; 31 Department of Surgery, Hospital Universitario Central de Asturias, 33011 Oviedo, Spain; 32 Department of Surgery, Hospital del Mar, 08003 Barcelona, Spain; 33 Department of Surgery, Hospital Universitario de Getafe, 28905 Getafe, Spain; 34 Department of Surgery, Hospital Universitario Arnau de Vilanova, 25198 Lleida, Spain; 35 Department of Surgery, Hospital Universitario Infanta Leonor, 28031 Madrid, Spain; 36 Department of Surgery, Hospital Universitario Morales Meseguer, 30008 Murcia, Spain; 37 Department of Surgery, Clínica La Luz, 28003 Madrid, Spain; 38 Department of Surgery, Hospital Universitario Puerta del Mar, 11009 Cádiz, Spain; 39 Department of Surgery, Hospital General Universitario de Ciudad Real, 13005 Ciudad Real, Spain; 40 Department of Surgery, Hospital General de Valencia, 46014 Valencia, Spain; 41 Department of Surgery, Hospital Royo Villanova, 50015 Zaragoza, Spain; 42 Department of Surgery, Hospital Clínico de San Carlos, 28040 Madrid, Spain; 43 Department of Surgery, Hospital Universitario de Elche, 03203, Alicante, Spain; 44 Department of Surgery, Hospital Universitario de Salamanca, 37007 Salamanca, Spain; 45 Department of Surgery, Hospital Clínico de Valladolid, 47007 Valladolid, Spain; 46 Instituto de Investigación Biosanitaria ibs.GRANADA, School of Medicine, University of Granada, 18016 Granada, Spain

**Keywords:** neuroendocrine tumors, outcomes, left pancreatectomy, pancreas, surgery

## Abstract

**Background:** Whether tumor functional status influences postoperative outcomes after left pancreatectomy (LP) for pancreatic neuroendocrine tumors (pNETs) remains unknown. We evaluated the association between functional status, perioperative management, and postoperative outcomes in a prospective nationwide cohort of patients undergoing LP for pNET. **Methods:** This prespecified analysis of the prospective multicenter SPANDISPAN study included consecutive patients undergoing elective LP at 41 Spanish hospitals between February 2022 and January 2023. Of 98 patients with pNET, functional status was available in 88 (14 functioning and 74 non-functioning tumors). Perioperative characteristics, spleen preservation, and postoperative outcomes were compared according to tumor functional status. **Results:** Spleen preservation was performed in 28.6% of functioning and 18.9% of non-functioning tumors (*p* = 0.472; risk difference 9.7 percentage points, 95% CI [confidence interval] −15.6 to 34.9). Patients with functioning tumors had lower rates of clinically relevant postoperative pancreatic fistula (7.1% vs. 36.5%; *p* = 0.032; risk difference −29.3 percentage points, 95% CI −46.7 to −12.0), shorter length of hospital stay (median 4 vs. 7 days; *p* = 0.036), and no 90-day readmissions (0% vs. 31.1%; *p* = 0.017; risk difference −31.1 percentage points, 95% CI −41.6 to −20.5). Tumor functional status was not significantly associated with the use of spleen-preserving resection. **Conclusions:** Functional status is associated with clinically relevant differences in postoperative outcomes after LP for pNET. Patients with functioning tumors experienced lower postoperative morbidity than those with non-functioning tumors, whereas no statistically significant association was detected between tumor functional status and spleen preservation. These exploratory findings suggest that tumor functional status may warrant consideration as an additional factor in perioperative risk stratification and patient counseling, pending confirmation in larger, adequately powered studies; spleen preservation should continue to be guided by established oncological criteria.

## 1. Introduction

Pancreatic neuroendocrine tumors (pNETs) are uncommon neoplasms, accounting for approximately 1–2% of all pancreatic tumors. However, their incidence has steadily increased over recent decades, largely owing to the widespread use of cross-sectional imaging, including contrast-enhanced computed tomography, which also plays a key role in the differential diagnosis of pNETs from other focal pancreatic lesions such as chronic mass-forming pancreatitis [1], and improved recognition of this disease entity. Consequently, the number of patients undergoing surgical treatment has risen, making pNETs the second most common indication for pancreatic resection after pancreatic ductal adenocarcinoma [2].

Pancreatic neuroendocrine tumors are classified as functioning or non-functioning neoplasms. Non-functioning tumors account for approximately 70–80% of cases and are often diagnosed incidentally, whereas functioning tumors represent about 20%. Therefore, whether these biological differences translate into differences in postoperative outcomes after left pancreatectomy (LP) remains uncertain.

The biological behavior of pNETs is highly heterogeneous, ranging from the relatively indolent course of insulinomas, with a malignancy risk of 5–10%, to other functioning tumors, in which the risk of malignant behavior may be as high as 60–90% [2]. Non-functioning tumors also carry malignant potential, with the risk of nodal or distant metastasis at diagnosis and of recurrence after resection increasing with tumor size and histological grade; consequently, long-term oncological follow-up is recommended regardless of functional status [3,4].

Because most pNETs arise in the pancreatic body and tail, LP is the standard surgical procedure for most patients. In carefully selected cases, particularly those with small, low-grade tumors, spleen-preserving LP (SPLP) has emerged as an attractive alternative to reduce long-term immunologic and hematologic consequences of splenectomy. Nevertheless, the optimal indications for splenic preservation remain controversial, particularly because of concerns about the adequacy of lymph node assessment and long-term oncologic outcomes [3,4,5,6,7,8].

The clinical relevance of pNETs has also increased in Spain. In the prospective multicenter SPANDISPAN (SPANish DIStal PANcreatectomy) study, which included 311 LP performed at 41 Spanish hospitals, pNETs were the most common pathological diagnosis, accounting for 31.5% of all procedures [6,7]. Despite this high prevalence, national evidence on the surgical management and postoperative outcomes of patients undergoing LP for pNETs remains limited and is largely based on retrospective single-center series.

To date, no prospective, nationwide, multicenter study has evaluated whether tumor functional status influences perioperative management and postoperative outcomes after LP for pNETs. Therefore, this study aimed to assess the influence of tumor functional status on perioperative management and postoperative outcomes in a prospective, nationwide, multicenter cohort of patients undergoing LP for pNETs. As a secondary objective, we analyzed current patterns of spleen preservation and identified factors associated with postoperative morbidity.

## 2. Materials and Methods

This was a prospective, multicenter, observational snapshot study. Consecutive adult patients undergoing elective LP at 41 participating Spanish centers from 1 February 2022, to 31 January 2023, were included. Eligible patients were aged 18 years or older and underwent elective LP for benign or malignant pancreatic lesions during the study period. Excluded were emergency procedures, LP with celiac axis resection, and resections performed after previous pancreatoduodenectomy.

The study was conducted in accordance with the 2013 Declaration of Helsinki and approved by the Institutional Research Ethics Committee of Health Department 19 (Reference CEIm: 2021-078). The manuscript was prepared in accordance with the STROBE recommendations for observational studies [9].

Participation was offered to Spanish hospitals that had previously collaborated on the Spanish Association of Surgeons/International Hepato-Pancreato-Biliary Association (AEC/IHPBA) National Survey on Pancreatic Surgery Units [10]. Data were prospectively recorded in a dedicated REDCap (Research Electronic Data Capture, Vanderbilt University, Nashville, TN, USA) system. All study variables had to be completed before patient inclusion to ensure data completeness. Written informed consent was obtained from all participants before enrollment, and postoperative complications were prospectively collected from medical and nursing records.

Preoperative variables included age, sex, body mass index (BMI), comorbidities, prior medical and surgical history, American Society of Anesthesiologists (ASA) physical status classification, and the Charlson Comorbidity Index [11]. Operative variables included surgical approach (open, laparoscopic, or robotic), conversion to open surgery, spleen preservation, spleen-preserving technique (Kimura or Warshaw), associated organ resection (defined as resection of at least one organ other than the spleen), estimated blood loss, and intraoperative blood transfusion.

Among the 14 functioning tumors, hormone-producing subtypes included 10 insulinomas, 2 somatostatinomas, and 2 glucagonomas. Functional status was assigned by the treating center based on the presence of a clinical hormonal syndrome corresponding to one of these categories, as recorded prospectively in the study case report form; granular biochemical confirmation data (e.g., specific hormone assay values) were not captured in the study database and therefore cannot be reported.

Postoperative outcomes included overall morbidity and 90-day mortality. Postoperative complications were graded using the Clavien–Dindo classification, with major complications defined as grade IIIa or higher [12]. Overall morbidity was also quantified using the Comprehensive Complication Index (CCI). Pancreas-specific complications, including postoperative pancreatic fistula, delayed gastric emptying, and postpancreatectomy hemorrhage, were defined according to the criteria established by the International Study Group of Pancreatic Surgery (ISGPS) [13,14,15].

Resection margins were classified according to the Royal College of Pathologists’ recommendations as R0 (tumor clearance ≥1 mm), R1 (tumor clearance <1 mm), and R2 (macroscopic residual disease) [16,17]. Pathological variables included tumor size, resection margin status, and pancreatic specimen length.

Reintervention was defined as any unplanned surgical, endoscopic, or radiologic procedure related to the index pancreatic operation. Length of hospital stay and 90-day readmission were also recorded. Participating centers were classified as high-volume institutions according to the criteria proposed by van der Heijde et al. [18].

Statistical analyses were conducted using IBM^®^ SPSS Statistics 28. Categorical variables are presented as frequencies and percentages, and continuous variables are reported as the median and interquartile range (IQR). Group comparisons were performed using the chi-square test or Fisher’s exact test for categorical variables and the Mann–Whitney U test for continuous variables. For the principal outcomes distinguishing functioning from non-functioning tumors, 95% confidence intervals for proportions and between-group risk differences were additionally calculated (Wilson score method for individual proportions; normal-approximation method for risk differences, with Haldane-Anscombe correction applied to odds ratios where a zero-count cell was present). No adjustment for multiple comparisons was applied; given the number of comparisons performed, results close to the *p* = 0.05 threshold should be interpreted with caution (see Section 4). A two-sided *p* value < 0.05 was considered statistically significant.

## 3. Results

Between February 2022 and January 2023, 313 patients underwent elective LP at 41 participating Spanish centers. pNETs were the underlying pathology in 98 patients (31.3%), and a minimally invasive approach was used in 217 procedures (69.3%). A detailed comparison of pNETs with other pathological indications for LP has been reported separately [6,7]. The present analysis is limited to the 98 patients with pNETs. Functional status was not recorded for 10 patients, generally reflecting incomplete completion of this case-report-form field at the participating site rather than a systematic reason for omission; these 10 patients were distributed across eight different hospitals, arguing against a single-center data-quality explanation. Compared with the 88 patients with functional status recorded, they did not differ significantly in age or sex, but had a significantly lower Charlson Comorbidity Index (median 2 vs. 4, *p* = 0.012) and a non-significant trend toward smaller tumors (median 13 vs. 20 mm, *p* = 0.069), so a degree of selection toward healthier patients with smaller lesions cannot be excluded. Functional status was available for 88 patients, who constituted the study population for subgroup analyses (Table 1).

**Table 1:** Comparison of patients with pancreatic neuroendocrine tumors and other pathological indications for left pancreatectomy in the prospective SPANDISPAN cohort.

Variables	Total	pNET		*p* Value
		No	Yes	
**Patients**	313	215 (68.6)	98 (31.3)	
**Age, years, median (IQR)**	65 (55–74)	67 (57–74)	62 (51–72)	**0.025**
**Gender, n (%)**				
Male	146 (46.6)	88 (40.9)	58 (59.2)	**0.003**
Female	167 (53.4)	127 (59.1)	40 (40.8)	
**ASA Physical Status, n (%)**				
I	17 (5.4)	11 (5.1)	6 (6.1)	0.842
II	149 (47.6)	103 (47.9)	46 (46.9)	
III	141 (45.0)	96 (44.7)	45 (45.9)	
IV	6 (1.9)	5 (2.3)	1 (1.0)	
**BMI, median (IQR)**	27.5 (24–30.1)	26.4 (23.5–29.6)	27.7 (23.3–31.1)	0.387
**Charlson Comorbidity Index**	4 (2–5)	4 (2–6)	4 (2–5)	0.482
**Comorbidities, n (%)**	237 (75.7)	163 (75.8)	74 (75.5)	0.954
Hypertension	128 (40.9)	92 (42.8)	36 (36.7)	0.312
Diabetes mellitus	87 (27.8)	61 (28.4)	26 (26.5)	0.736
COPD	24 (7.7)	16 (7.4)	8 (8.2)	0.824
Ischemic heart disease	17 (5.4)	12 (5.6)	5 (5.1)	0.862
Cerebrovascular disease	14 (4.5)	12 (5.6)	2 (2.0)	0.160
Liver disease	13 (4.2)	8 (3.7)	5 (5.1)	0.570
Chronic kidney disease	16 (5.1)	9 (4.2)	7 (7.1)	0.271
**Past surgical history, n (%)**	139 (44.4)	102 (47.4)	37 (37.8)	0.110
**Tumor location, n (%)**				
Tail	127 (40.6)	78 (36.3)	49 (50.0)	0.094
Body	86 (27.5)	61 (28.4)	25 (25.5)	
Body-tail	77 (24.6)	60 (27.9)	17 (17.3)	
Neck	23 (7.3)	16 (7.4)	7 (7.1)	
**Tumor characteristics, n (%)**				
Associated organ involvement	37 (11.8)	28 (13.0)	9 (9.2)	0.329
Preoperative biopsy	183 (58.5)	123 (57.2)	60 (61.2)	0.504
Tumor size (mm)	28 (17–44)	30 (20–47)	20 (13–36)	**<0.001**
Pancreatic duct size (mm)	2 (2–5)	3 (2–5)	3 (1.7–4)	0.119
**Hospital volume, n (%)**				
High (≥10 patients)	146 (46.6)	100 (46.5)	46 (46.9)	0.944
Low (<10 patients)	167 (53.4)	115 (53.5)	52 (53.1)	
**Operative variables, n (%)**				
Minimally invasive approach	217 (69.3)	137 (63.7)	80 (81.6)	**0.001**
Spleen preservation	42 (13.4)	23 (10.7)	19 (19.4)	**0.036**
Associated organ resection	42 (13.4)	33 (15.3)	9 (9.2)	0.138
Vascular resection	10 (3.2)	9 (4.2)	1 (1.0)	0.140
Drain insertion	282 (90.1)	196 (91.2)	86 (87.8)	0.349
Use of sealant	89 (28.4)	64 (29.8)	25 (25.5)	0.439
Intraoperative pRBC transfusion	20 (6.4)	16 (7.4)	4 (4.1)	0.260
Estimated blood loss (mL)	120 (50–300)	120 (100–300)	135 (50–300)	0.613
Operative time (min)	240 (180–300)	240 (180–300)	239 (180–288)	0.367
**Postoperative outcomes, n (%)**				
Postoperative somatostatin use	99 (31.6)	55 (74.4)	44 (44.9)	**<0.001**
Delayed gastric emptying	9 (6.5)	5 (2.3)	4 (4.1)	0.469
**Pancreatic fistula, n (%)**				
No POPF	193 (61.7)	140 (65.1)	53 (54.1)	**0.017**
Biochemical leak	58 (18.5)	42 (19.5)	16 (16.3)	
Clinically relevant POPF (ISGPS grade B/C)	62 (19.8)	33 (15.3)	29 (29.6)	
Post-pancreatectomy hemorrhage	17 (5.4)	14 (6.5)	3 (3.1)	0.286
Major complications (Clavien–Dindo ≥ IIIa)	67 (21.4)	47 (21.9)	20 (20.4)	0.771
Comprehensive Complication Index (CCI)	0 (8.7–20.9)	8.7 (0–20.9)	8.7 (0–20.9)	0.643
Length of stay, days, median (IQR)	7 (5–9)	7 (5–10)	7 (4–9)	0.186

Note: Length-of-stay values in this row (Total, pNET: No, and pNET: Yes columns) were recalculated directly from the study database and are consistent with the functioning/non-functioning breakdown reported in Table 2 (4 vs. 7 days). BMI, Body Mass Index; ASA, American Society of Anaesthesiologists; POPF, postoperative pancreatic fistula; ISGPS, International Study Group of Pancreatic Surgery. Bold values are the statistically significant data (*p* < 0.05).

**Table 2:** Comparison of perioperative characteristics and postoperative outcomes according to tumor functional status in patients with pancreatic neuroendocrine tumors.

Variable	Tumor Functional Status		*p* Value	95% CI (Functioning vs. Non-Functioning)
	Functioning (n = 14)	Non-Functioning (n = 74)		
**Age, years, median (IQR)**	52 (39–62)	63 (55–74)	**0.016**	
**Gender, n (%)**				
Male	5 (35.7)	48 (64.9)	**0.041**	
Female	9 (64.3)	26 (35.1)		
**BMI, median (IQR)**	27 (23–31)	28 (23–32)	0.673	
**Charlson Comorbidity Index**	2.5 (2–5)	5 (2–6)	**0.022**	
**ASA Physical Status, n (%)**				
I	2 (14.3)	4 (5.4)	0.520	
II	7 (50.0)	31 (41.9)		
III	5 (35.7)	38 (51.4)		
IV	0 (0.0)	1 (1.4)		
**Any comorbidity, n (%)**	10 (71.4)	60 (81.1)	0.472	
Preoperative hypertension	5 (35.7)	29 (39.2)	0.807	
Preoperative diabetes mellitus	0 (0.0)	24 (32.4)	**0.009**	
Preoperative COPD	0 (0.0)	8 (10.8)	0.346	
Ischemic heart disease	0 (0.0)	5 (6.8)	0.411	
Cerebrovascular disease	0 (0.0)	2 (2.7)	0.706	
Liver disease	0 (0.0)	5 (6.8)	0.411	
Chronic kidney disease	0 (0.0)	7 (9.5)	0.591	
**Abdominal surgical history, n (%)**	4 (28.6)	31 (41.9)	0.350	
**Previous pancreatic surgery, n (%)**	0 (0.0)	2 (2.7)	0.706	
**Preoperative symptoms, n (%)**	11 (78.6)	27 (36.5)	**0.004**	
Abdominal pain	0 (0.0)	18 (24.3)	0.064	
**Tumor location, n (%)**				
Tail	5 (35.7)	39 (52.7)	0.388	
Body	3 (21.4)	20 (27.0)		
Body-tail	4 (28.6)	10 (13.5)		
Neck	2 (14.3)	5 (6.8)		
**Associated organ involvement, n (%)**	1 (7.1)	7 (9.5)	0.626	
**Preoperative biopsy, n (%)**	5 (35.7)	50 (67.6)	**0.024**	
**Tumor size (mm)**	17 (14–23)	23 (13–37)	0.424	
**Pancreatic duct size (mm)**	3 (1–4)	3 (1.7–4)	0.837	
**Pancreatic gland texture, soft, n (%)**	8 (57.1)	53 (71.6)	0.346	
**Resection margin, R0, n (%)**	13 (92.9)	71 (95.9)	0.507	
**Lymph nodes resected, median (IQR)**	1 (0–5)	6 (2–11)	**0.016**	
**High-volume center, n (%)**	7 (50.0)	34 (45.9)	0.780	
**Minimally invasive approach, n (%)**	14 (100.0)	57 (77.0)	0.063	RD +23.0 pp (13.4 to 32.6); OR 8.8 (0.50–155.6)
**Spleen preservation, n (%)**	4 (28.6)	14 (18.9)	0.472	RD +9.7 pp (−15.6 to 34.9); OR 1.71 (0.47–6.27)
**Associated organ resection, n (%)**	0 (0.0)	8 (10.8)	0.346	
**Vascular resection, n (%)**	0 (0.0)	1 (1.4)	0.841	
**Surgical drains, n (%)**	13 (92.9)	66 (89.2)	0.563	
**Conversion, n (%)**	2 (14.3)	7 (12.3)	0.842	
**Use of sealant, n (%)**	3 (21.4)	21 (28.4)	0.750	
**Intraoperative transfusion, n (%)**	2 (14.3)	2 (2.7)	0.117	
**Estimated blood loss (mL)**	100 (10–300)	150 (50–300)	0.200	
**Operative time (min)**	180 (158–240)	240 (180–300)	0.117	
**Postoperative somatostatin use, n (%)**	5 (35.7)	34 (45.9)	0.480	
**Major complications (Clavien-Dindo ≥ IIIa)**	1 (7.1)	19 (25.7)	0.175	RD −18.5 pp (−35.3 to −1.8); OR 0.22 (0.03–1.82)
**Comprehensive Complication Index (CCI)**	0 (0–8.7)	8.7 (0–26.2)	**0.042**	
**POPF, any grade, n (%)**	3 (21.4)	39 (52.7)	**0.032**	RD −31.3 pp (−55.6 to −7.0); OR 0.24 (0.06–0.95)
**Clinically relevant POPF (ISGPS grade B/C), n (%)**	1 (7.1)	27 (36.5)	**0.032**	RD −29.3 pp (−46.7 to −12.0); OR 0.13 (0.02–1.08)
**Delayed gastric emptying, n (%)**	0 (0.0)	4 (5.4)	0.493	
**Post-pancreatectomy hemorrhage, n (%)**	0 (0.0)	3 (4.1)	0.591	
**Reintervention, n (%)**	1 (7.1)	7 (9.5)	0.626	
**Length of stay, days, median (IQR)**	4 (3–7)	7 (5–9)	**0.036**	
**90-day readmission, n (%)**	0 (0.0)	23 (31.1)	**0.017**	RD −31.1 pp (−41.6 to −20.5)
**Exocrine insufficiency, n (%)**	1 (7.1)	12 (16.2)	0.683	
**90-day mortality, n (%)**	0 (0.0)	1 (1.4)	0.841	

BMI, Body Mass Index; ASA, American Society of Anaesthesiologists; POPF, postoperative pancreatic fistula; ISGPS, International Study Group of Pancreatic Surgery; RD, risk difference (percentage points, functioning minus non-functioning); OR, odds ratio (Haldane–Anscombe corrected where a zero-count cell was present). A 95% confidence interval (CI) column is provided for the principal outcomes distinguishing functioning from non-functioning tumors; confidence intervals for median-based continuous outcomes (length of stay, CCI) would require patient-level data. Bold values are the statistically significant data (*p* < 0.05).

### 3.1. Functioning Versus Non-Functioning Tumors: Baseline Characteristics

Among 88 patients with available functional status, 14 tumors (15.9%) were functioning and 74 (84.1%) were non-functioning (Table 2). Patients with functioning tumors were significantly younger (median 52 vs. 63 years, *p* = 0.016) and more often female (64.3% vs. 35.1% male, *p* = 0.041) than those with non-functioning tumors. Functioning tumors were also associated with a lower Charlson Comorbidity Index (2.5 vs. 5, *p* = 0.022) and a markedly lower prevalence of preoperative diabetes mellitus (0% vs. 32.4%, *p* = 0.009). As expected, preoperative symptoms were far more common in patients with functioning tumors (78.6% vs. 36.5%, *p* = 0.004), whereas preoperative biopsies were performed less often in this group (35.7% vs. 67.6%, *p* = 0.024). Tumor size did not differ significantly between subgroups by conventional testing (median 17 vs. 23 mm, *p* = 0.424; range 11–90 mm in functioning vs. 3–121 mm in non-functioning tumors), although the absence of a significant difference in medians in this small subgroup does not exclude a contribution of tumor size to the observed outcome differences (see Section 4 and sensitivity analysis below). Pancreatic duct diameter (median 3 mm in both groups, *p* = 0.837), tumor location, and center volume category did not differ significantly between subgroups.

### 3.2. Surgical Approach and Spleen Preservation According to Functional Status

A minimally invasive approach was used in all 14 patients with functioning tumors (100%) compared with 77.0% of those with non-functioning tumors, a difference that did not reach statistical significance (*p* = 0.063; risk difference +23.0 percentage points, 95% CI 13.4–32.6; OR 8.8, 95% CI 0.50–155.6). Given the small sample size, this comparison is underpowered, and the numerically large difference should not be interpreted as evidence that the two surgical approaches were used comparably according to functional status. Spleen preservation was attempted in 4 of 14 patients (28.6%) with functioning tumors and in 14 of 74 patients (18.9%) with non-functioning tumors; no statistically significant association with tumor functional status was detected (*p* = 0.472; risk difference +9.7 percentage points, 95% CI −15.6 to 34.9; OR 1.71, 95% CI 0.47–6.27). Associated organ resection, vascular resection, operative time, estimated blood loss, use of hemostatic sealants, and intraoperative transfusion were also comparable between the two groups.

### 3.3. Postoperative Outcomes According to Functional Status

Postoperative outcomes according to tumor functional status are summarized in Table 2. Postoperative pancreatic fistula (POPF) of any grade occurred in 21.4% of patients with functioning tumors vs. 52.7% of those with non-functioning tumors (*p* = 0.032; risk difference −31.3 percentage points, 95% CI −55.6 to −7.0; OR 0.24, 95% CI 0.06–0.95), and clinically relevant (ISGPS grade B/C) POPF occurred in 7.1% vs. 36.5%, respectively (*p* = 0.032; risk difference −29.3 percentage points, 95% CI −46.7 to −12.0; OR 0.13, 95% CI 0.02–1.08). Median length of hospital stay was shorter after resection of functioning tumors (4 vs. 7 days, *p* = 0.036), and the Comprehensive Complication Index was lower (median 0 vs. 8.7, *p* = 0.042). No patient with a functioning tumor required 90-day readmission, compared with 31.1% of patients with non-functioning tumors (*p* = 0.017; risk difference −31.1 percentage points, 95% CI −41.6 to −20.5). Major morbidity (Clavien–Dindo ≥ IIIa), delayed gastric emptying, post-pancreatectomy hemorrhage, reintervention, and 90-day mortality did not differ significantly between subgroups, and no patient with a functioning tumor died. The postoperative use of somatostatin analogs was similar between groups (35.7% versus 45.9%, *p* = 0.480).

As a sensitivity analysis addressing reviewer concerns about confounding by baseline imbalances, we fitted a multivariable logistic regression model for the primary outcome of clinically relevant (grade B/C) POPF, adjusting for age, preoperative diabetes mellitus, and tumor size. Functioning tumor status remained associated with lower odds of clinically relevant POPF after adjustment (adjusted OR 0.15, 95% CI 0.02–1.31; *p* = 0.087), directionally consistent with, though attenuated relative to, the unadjusted estimate, with a wide confidence interval reflecting the small functioning-tumor subgroup. Given the very small number of events, adjusted models were not attempted for the remaining outcomes; 90-day readmission, in particular, had zero events in the functioning group, precluding regression-based adjustment.

## 4. Discussion

The principal finding of this nationwide prospective study is that tumor functional status is associated with clinically meaningful differences in postoperative outcomes after LP for pNET, although—as detailed in the Limitations below—this unadjusted association is likely confounded and should not be interpreted as an independent effect of functional status itself. This study addresses a different question from our group’s previous analysis of the same national cohort, which compared postoperative outcomes across histological indications for LP, including pNETs, adenocarcinoma, IPMN/MCN, and other lesions [6,7]. By restricting the analysis to patients with pNET and stratifying them by tumor functional status, we evaluated whether functioning and non-functioning tumors differ in perioperative management and short-term postoperative outcomes. To our knowledge, this is the first prospective nationwide multicenter study specifically addressing this question. These findings provide the basis for a more individualized approach to risk stratification in patients with pNET.

The most relevant finding of our study is that patients undergoing LP for a functioning pNET had a substantially more favorable postoperative course than those operated on for a non-functioning tumor, with a lower rate of clinically relevant POPF (7.1% vs. 36.5%), a shorter hospital stay, a lower Comprehensive Complication Index, and no 90-day readmissions. Although functioning tumors were numerically smaller, this difference did not reach statistical significance (median 17 vs. 23 mm, *p* = 0.424), suggesting that tumor size alone does not fully explain the observed differences in postoperative outcomes (Figure S1). One possible, unproven explanation is earlier diagnosis driven by hormonal symptoms, present in 78.6% of patients with functioning tumors compared with 36.5% of those with non-functioning disease: earlier diagnosis could plausibly lead to surgery for smaller, more favorable lesions, and might reflect differences in pancreatic parenchymal characteristics at the time of surgery, but this mechanism was not directly assessed in our dataset and remains speculative. The younger age and lower comorbidity burden of patients with functioning tumors may also have contributed to their more favorable recovery, and—together with the other baseline imbalances described in the Limitations—likely confound this unadjusted comparison. In a sensitivity analysis adjusting for age, diabetes, and tumor size, the association between functional status and clinically relevant POPF persisted numerically but with a wide confidence interval that approached unity (see Results), consistent with a real but imprecisely estimated effect, or with residual confounding that this small sample cannot exclude; this adjusted estimate should be interpreted as hypothesis-generating rather than confirmatory.

To our knowledge, a direct comparison of POPF rates and other perioperative outcomes between functioning and non-functioning pNETs undergoing LP has not been reported at this scale. This finding, although it requires confirmation in larger series given the small number of functioning tumors in our cohort (n = 14), may have implications for preoperative risk stratification and patient counseling.

The higher risk of clinically relevant POPF observed among patients with non-functioning pNETs aligns with the broader literature comparing pNETs with other pancreatic pathologies. Two recent large, nationwide analyses using the composite “Ideal Outcome” metric found that pNETs, as a group, carry a higher risk of clinically relevant POPF than pancreatic ductal adenocarcinoma, yet this translated into a lower overall rate of Ideal Outcome [19,20]. Although these studies did not distinguish between functioning and non-functioning pNETs, they support the concept that POPF risk is not uniform across pancreatic pathologies. Our data suggest that within the pNET population, this excess fistula risk is concentrated among non-functioning tumors. A biologically plausible explanation is that clinically relevant POPF is more common in patients with established pancreatic risk factors, including a soft gland and a small pancreatic duct. In our cohort, pancreatic gland texture, resection margin status, and lymph node yield were available and are reported in Table 2. Gland texture did not differ significantly between groups (soft gland in 57.1% of functioning vs. 71.6% of non-functioning tumors, *p* = 0.346), and pancreatic duct diameter was also similar (median 3 mm in both, *p* = 0.837); although not statistically significant, the numerically lower proportion of soft glands in the functioning group is directionally consistent with their lower POPF rate. Dynamic perioperative platelet count trajectories have also been proposed as an early biomarker of POPF risk after pancreatic resection; this variable was not collected in the SPANDISPAN case report form and could not be examined in the present analysis, but represents a promising avenue for future prospective studies in this population. Notably, patients with functioning tumors had a markedly lower lymph node yield than those with non-functioning tumors (median 1 vs. 6, *p* = 0.016), suggesting that functioning tumors were more often managed with a less extensive lymphadenectomy (or parenchyma-sparing resection); this difference in surgical extent is a plausible additional contributor to the observed differences in POPF, length of stay, and readmission, and should be regarded as a further source of confounding rather than as evidence of a direct biological effect of functional status itself (see Limitations).

Regarding our secondary objective, we found no statistically significant association between tumor functional status and the decision to preserve the spleen during LP for pNET. Spleen preservation was attempted in a numerically higher, though not statistically significant, proportion of functioning tumors (28.6% vs. 18.9%; *p* = 0.472; risk difference +9.7 percentage points, 95% CI −15.6 to 34.9). This absence of a statistically significant difference does not establish equivalence between the two groups and should not be over-interpreted given the small functioning subgroup; it is nonetheless consistent with, and does not contradict, the hypothesis that spleen preservation was guided by established oncological and technical criteria rather than by tumor functional status, for which no biological rationale currently exists. This aligns with current surgical practice, in which the decision to preserve the spleen is primarily determined by tumor size, proximity to the splenic vessels, and the need for formal lymphadenectomy, rather than by endocrine function. A larger, adequately powered study would be needed to determine definitively whether functional status truly has no influence on the choice of spleen-preserving resection.

The available evidence is consistent with this approach. A recent systematic review and meta-analysis found that spleen-preserving LP for pNET was associated with less blood loss, fewer major complications, and a shorter hospital stay than splenectomy, without compromising R0 resection or lymph node yield [8]. Likewise, a large multicenter European study found no oncological benefit of splenectomy for overall or recurrence-free survival in non-functioning pNET and supported the Warshaw technique as a safe alternative whenever lymphadenectomy is indicated [21]. Collectively, these findings reinforce current ENETS recommendations that spleen preservation should be considered whenever oncologically appropriate, irrespective of tumor functional status [3,4].

Our findings suggest that patients undergoing LP for a non-functioning pNET should be counseled about a substantially higher risk of clinically relevant POPF, longer hospital stays, and readmission than those undergoing resection for a functioning tumor, bearing in mind that this counseling reflects an observed association from an unadjusted, confounded comparison rather than a demonstrated causal or independent effect of functional status. Tumor functional status may therefore be an additional factor in perioperative risk stratification, regardless of whether the spleen is preserved. These findings may justify a lower threshold for fistula-mitigation strategies, closer postoperative surveillance, and more cautious drain and discharge management in patients with non-functioning pNETs.

Looking beyond the anatomical and clinical parameters assessed here, functional information may in the future be combined with multimodal imaging for more comprehensive preoperative tumor characterization and treatment planning; advances in the integration of nuclear medicine with complementary imaging modalities may support this direction [22].

This study has several limitations. First, as with any observational snapshot study, we cannot exclude residual confounding by unmeasured variables. Indeed, patients with functioning tumors differed from those with non-functioning tumors not only in the outcomes reported above but also at baseline—they were significantly younger, had a lower Charlson Comorbidity Index, a lower prevalence of diabetes, and more frequently presented with symptoms; the higher comorbidity burden and diabetes prevalence in the non-functioning group are themselves likely related, at least in part, to their older age. In addition, a minimally invasive approach was used in 100% of functioning-tumor resections versus 77.0% of non-functioning-tumor resections; because a minimally invasive approach is itself associated with shorter length of stay and a lower Comprehensive Complication Index, this difference in surgical approach should be regarded as a further potential confounder of the length-of-stay and CCI comparisons, alongside functional status itself. Each of these factors is independently associated with postoperative morbidity, length of stay, and readmission after pancreatic surgery, and multivariable adjustment was not feasible given the small number of events in the functioning-tumor subgroup (see below). The associations reported here should therefore not be interpreted as an independent effect of tumor functional status, but as an unadjusted, confounded comparison that is hypothesis-generating for future, adequately powered studies.

Second, our dataset does not include the Ki-67 proliferation index or WHO tumor grade, precluding assessment of the relationship between tumor grade and either functional status or postoperative outcomes. Pancreatic gland texture, resection margin status, and lymph node yield were available in the database and have been incorporated into Table 2 and the Section 4 above; pancreatic duct diameter was also recorded and did not differ significantly between groups (median 3 mm in both, *p* = 0.837). Even so, the proposed biological explanation for the lower POPF rate among functioning tumors remains only partly supported by these variables (see Section 4) and should still be regarded as hypothesis-generating, particularly given the small functioning-tumor subgroup and the observed difference in lymph node yield between groups, which suggests differing extents of resection as an additional, unmeasured source of confounding.

Third, functional status was available for only 88 of the 98 patients with pNET, and the functioning subgroup was particularly small (n = 14). Although several univariable comparisons reached statistical significance, the number of events was insufficient to support multivariable analysis; exact 95% confidence intervals for the principal outcomes (Table 2) illustrate the resulting imprecision, with several intervals approaching or crossing the null. In addition, no correction for multiple comparisons was applied across the numerous baseline and outcome comparisons reported in Table 1 and Table 2; several statistically significant findings (e.g., the Comprehensive Complication Index, *p* = 0.042; length of stay, *p* = 0.036; and the borderline non-significant comparison for minimally invasive approach, *p* = 0.063) lie close to the conventional significance threshold and should be regarded as hypothesis-generating rather than confirmatory. Furthermore, pooling different functioning-tumor subtypes (e.g., insulinoma, gastrinoma, and other hormonal syndromes) may introduce additional clinical and biological heterogeneity that this small subgroup was not powered to explore separately.

Fourth, the one-year recruitment window, while allowing a genuinely prospective, contemporary snapshot of national practice, does not permit assessment of long-term oncological outcomes such as recurrence or survival, which are particularly relevant to spleen preservation policy. Finally, because this cohort was drawn exclusively from Spanish centers, the generalizability of these findings to healthcare systems with different referral patterns or surgical volumes should be interpreted with caution.

## 5. Conclusions

In conclusion, in this prospective nationwide Spanish cohort of patients undergoing left pancreatectomy for pNET, functioning tumors were associated with more favorable postoperative outcomes than non-functioning tumors, including lower rates of clinically relevant postoperative pancreatic fistula, shorter hospital stays, and fewer readmissions, in an unadjusted comparison that did not account for baseline differences between groups (see Limitations). By contrast, no statistically significant association was detected between spleen-preserving resection and tumor functional status, suggesting that this technical decision should continue to be guided by established oncological and anatomical criteria. These exploratory findings suggest that tumor functional status may warrant consideration as an additional factor in perioperative risk stratification and patient counseling, pending confirmation in larger, adequately powered studies with adjustment for baseline confounders; current recommendations that spleen preservation be considered whenever oncologically appropriate are reinforced.

## Data Availability

The datasets used and/or analyzed during the current study are available from the corresponding author on reasonable request.

## Supplementary Materials

The following supporting information can be downloaded at: https://www.mdpi.com/article/10.3390/jcm15187035/s1, Figure S1: Distribution of tumor size by functional status.

## Abbreviations

AEC/IHPBA: Spanish Association of Surgery/International Hepato-Pancreato-Biliary Association; ASA: American Society of Anesthesiologists; CCI: Comprehensive Complication Index; IQR: Interquartile range; ISGPS: International Study Group on Pancreatic Surgery; LP: Left pancreatectomy; pNET: pancreatic Neuroendocrine Tumor; POPF: Postoperative pancreatic fistula; SPANDISPAN: SPANish DIStal PANcreatectomy; SPLP: spleen-preserving LP.
