# Supplementary material for: Functional Status and Postoperative Outcomes After Left Pancreatectomy for Pancreatic Neuroendocrine Tumors: An Exploratory Analysis from the Prospective SPANDISPAN Study
Source: J Clin Med. 2026 Sep 11;15(18):7035. doi: 10.3390/jcm15187035 (PMC13607226; doi:10.3390/jcm15187035)
Supplement: Supplementary file 1 [file jcm-15-07035-s001.zip › jcm-4525004-supplementary.pdf]

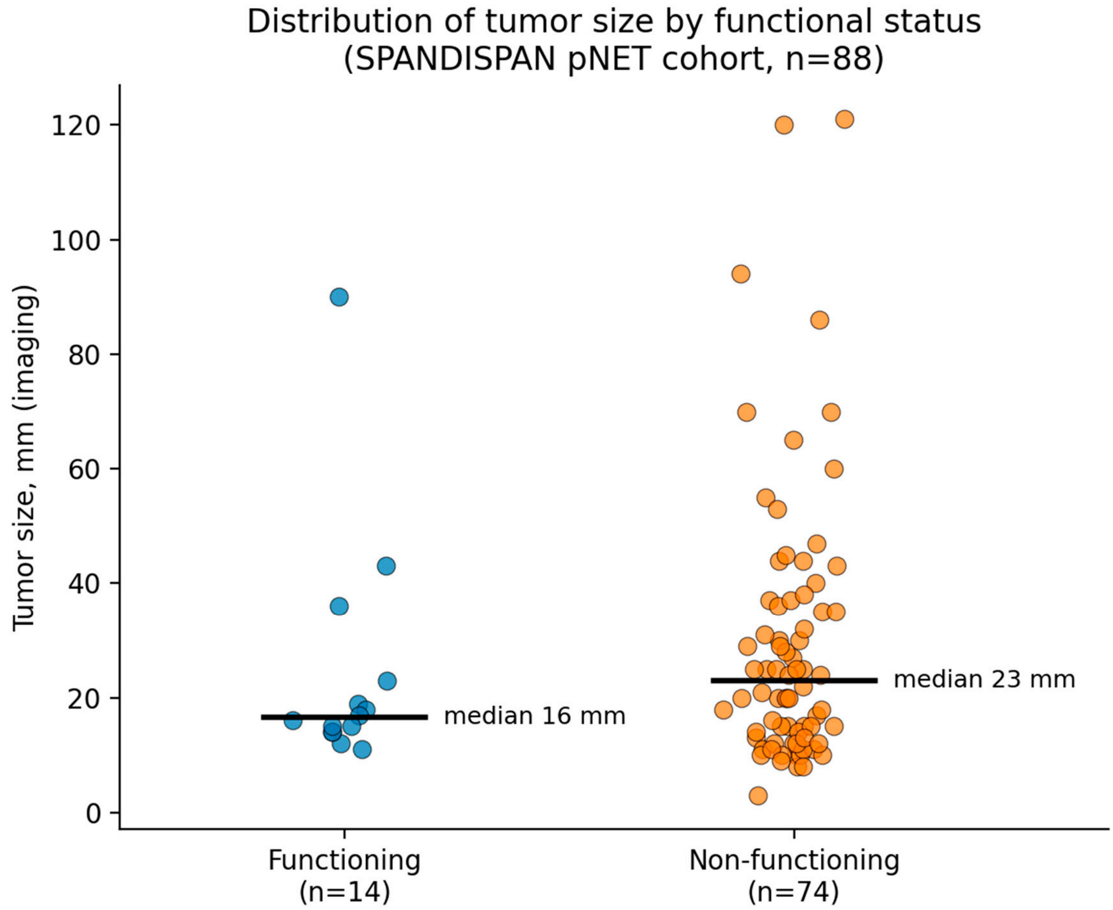

Figure S1. Distribution of tumor size by functional status.
